# Using the Web to Collect Data on Sensitive Behaviours: A Study Looking at Mode Effects on the British National Survey of Sexual Attitudes and Lifestyles

**DOI:** 10.1371/journal.pone.0147983

**Published:** 2016-02-11

**Authors:** Sarah Burkill, Andrew Copas, Mick P. Couper, Soazig Clifton, Philip Prah, Jessica Datta, Frederick Conrad, Kaye Wellings, Anne M. Johnson, Bob Erens

**Affiliations:** 1 Research Department of Infection and Population Health, University College London, London, United Kingdom; 2 Department of Medicine Solna, Karolinska Institutet, Stockholm, Sweden; 3 Institute for Social Research, University of Michigan, Ann Arbor, United States of America; 4 Department of Social and Environmental Research, London School of Hygiene and Tropical Medicine, London, England; 5 Department of Health Services Research & Policy, London School of Hygiene and Tropical Medicine, London, England; University of São Paulo, BRAZIL

## Abstract

**Background:**

Interviewer-administered surveys are an important method of collecting population-level epidemiological data, but suffer from declining response rates and increasing costs. Web surveys offer more rapid data collection and lower costs. There are concerns, however, about data quality from web surveys. Previous research has largely focused on selection biases, and few have explored measurement differences. This paper aims to assess the extent to which mode affects the responses given by the same respondents at two points in time, providing information on potential measurement error if web surveys are used in the future.

**Methods:**

527 participants from the third British National Survey of Sexual Attitudes and Lifestyles (Natsal-3), which uses computer assisted personal interview (CAPI) and self-interview (CASI) modes, subsequently responded to identically-worded questions in a web survey. McNemar tests assessed whether within-person differences in responses were at random or indicated a mode effect, i.e. higher reporting of more sensitive responses in one mode. An analysis of pooled responses by generalized estimating equations addressed the impact of gender and question type on change.

**Results:**

Only 10% of responses changed between surveys. However mode effects were found for about a third of variables, with higher reporting of sensitive responses more commonly found on the web compared with Natsal-3.

**Conclusions:**

The web appears a promising mode for surveys of sensitive behaviours, most likely as part of a mixed-mode design. Our findings suggest that mode effects may vary by question type and content, and by the particular mix of modes used. Mixed-mode surveys need careful development to understand mode effects and how to account for them.

## Introduction

Since the 1990’s there has been a decline in response rates, and an increase in costs of conducting interviewer-administered probability sample surveys,[1–3] which traditionally provide key epidemiological population data. Researchers and commissioners are increasingly seeking more cost-effective methods, particularly given current pressures on research budgets, and web surveys have become an attractive option given their relative low cost and quick turnaround times.[4,5] Initial concerns about biases in access to the internet have decreased over time, given high rates of coverage in European and other developed countries.[6] However, other concerns remain about data quality, including often very low response rates and response bias,[7,8] and the effect that mode of questionnaire administration (e.g. web compared with telephone or face-to-face interviews) may have on participants’ responses, referred to as ‘mode effect’. Researching how mode interacts with respondents’ propensity to answer in a certain way is important, since technological change and innovation often lead to new developments in data collection methods. Previously, this involved switching from paper questionnaires to computer-assisted methods, and currently it involves a shift from traditional postal or interviewer-administered surveys to web surveys. Web surveys are now the norm in market research,[7,9] but are less commonly used for academic and government studies of the general population. The move to increasing use of the web for research can involve collecting all data via a web survey or, perhaps more likely for academic research, by mixing modes within a study, and encouraging as many respondents as possible to complete the survey online in order to reduce costs. For example, using the web as part of a mixed mode design is attracting increasing interest for panel and cohort surveys which can mix modes either within or between waves of data collection.[10–13]

Previous research has highlighted that, even when identical questions are asked, different modes (and different samples) may provide discrepant answers,[14–16] and there may be potential advantages for data quality of using self-administered modes on computer, including web surveys.[17] For example, one benefit is the greater perceived anonymity when using self-administered modes, as this can result in higher reports of socially censured events (e.g. drug use) or of sensitive (e.g. sexual) behaviours,[18–20] and consequently potentially more accurate data when researching sensitive issues.

Most previous research on this topic has compared web and interviewer-administered surveys using different samples.[12,17,21,22] With this approach, however, it is difficult to disentangle mode effects from other impacts (e.g. differences in sample composition) on differences in estimates. This paper describes results from a study which asked respondents identically worded questions using two modes of data collection in order to assess within-person change. We investigate the impact of mode on responses for sensitive behaviours in order to examine the potential consequences of altering or mixing modes if the web is to be used either as a replacement for, or together with, a traditional interview survey.

## Methods

### Data

Our experiment used the third National Survey of Sexual Attitudes and Lifestyles (Natsal-3), a national probability sample survey of 15,162 men and women aged 16–74 resident in private households in Britain. Details of the survey methodology are published elsewhere.[23,24] Interviewers first asked questions, covering health conditions, learning about sex and first heterosexual experience, using a computer-assisted personal interview (CAPI). Eligible respondents were handed the laptop to read and answer the most sensitive questions themselves (e.g. on number of sexual partners, sexual practices, etc), referred to as computer-assisted self-interview (CASI). While the interviewer did not see the responses, s/he was present in the room during completion. The final attitude and socio-demographic modules were asked of everyone in CAPI. The response rate to Natsal-3 was 57.7% and the co-operation rate (i.e. of all eligible addresses contacted) was 65.8%.

Over the fieldwork period (September 2010-August 2012), the sample was issued in 8 ‘waves’, each wave representative of the population. Respondents from waves 7 and 8 (March-August 2012), were eligible for the follow-up web survey, roughly 1–2 months after their Natsal-3 interview. The web questionnaire included a sub-set of about 130 identically worded Natsal-3 questions.

**Fig 1:**
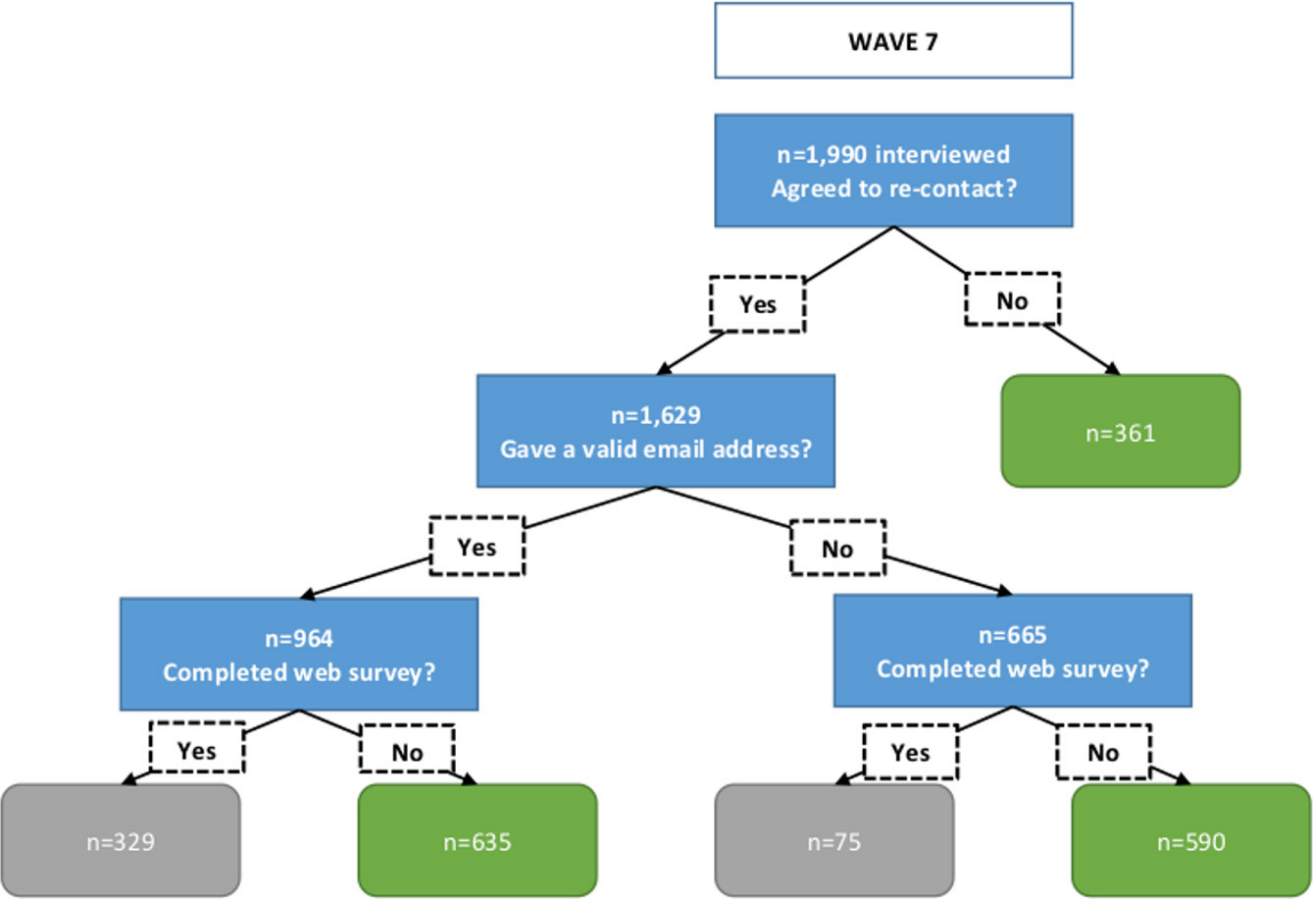
Sampling and response to web survey for Natsal-3 wave 7 respondents.

**Fig 2:**
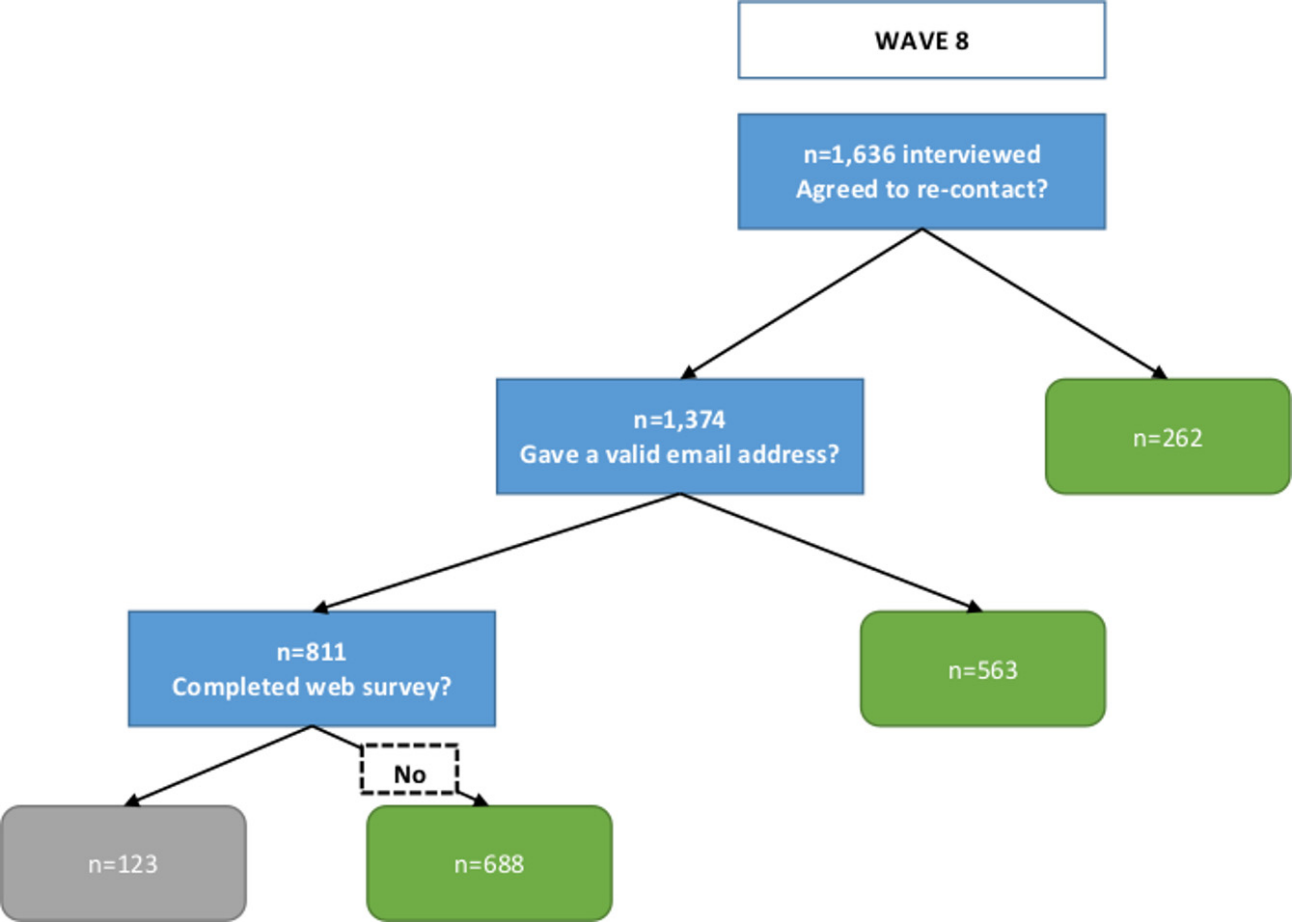
Sampling and response to web survey for Natsal-3 wave 8 respondents.

All wave 7 respondents who agreed to re-contact (n = 1629) were posted an invitation to undertake the web survey. An email invitation was also sent to those who provided a valid email address (n = 964), and an email reminder was sent after two weeks (no reminder was sent to the 665 respondents who had not provided an email). The web survey was completed by 404 wave 7 respondents. In order to boost numbers, the invitation was extended to wave 8 respondents, but only to those who agreed to re-contact *and* provided an email address (n = 811). They were invited by email only and no reminders were sent; 123 wave 8 respondents completed the web survey. A conditional £5 ‘token of appreciation’ was offered. Figs 1 and 2 represent the sampling process and response.

### Ethics statement

All Natsal-3 participants were given an information leaflet which they were able to discuss with the interviewer prior to participation. Consent was gained verbally, with the interviewer required to confirm in the computer programme that the respondent had read the information leaflet and agreed to participate before commencing the interview. The Natsal-3 study was approved by the Oxfordshire Research Ethics Committee A (reference: 09/H0604/27). Each participant consented to their own participation aside from the 16 and 17 year olds living at home, for whom a parent/guardian provided additional verbal consent for participation.

### Analysis

Responses to 7 demographic and 31 behavioural and opinion questions were examined. They were selected to cover a range of key measures included in the survey and were consistent with those examined in an earlier paper on whether web panel surveys could provide comparable estimates to Natsal-3.[25] All variables were coded as binary, and presented in a ‘yes/no’ format for consistency and ease of presentation.

Before viewing the results, we selected the response for each question we thought was likely to be more sensitive and potentially more susceptible to social desirability bias (e.g. the percentage reporting having taken illegal drugs rather than the percentage who have not). For reported number of sexual partners, we hypothesised that the extremes of the distributions, particularly for lifetime partners, would be the most sensitive responses,[26] and therefore examined reporting of 0 lifetime partners. We also examined a relative change between modes of 10% or more in reported number of (opposite-sex) lifetime partners, partners in the past 5 years and in the past year. Because of the small number of respondents reporting same-sex partners, we only looked at reports of ever having same-sex experience. We acknowledge that the sensitivity of different responses and mode effects may differ between men and women, a finding of past research.[27]

We compared responses between surveys for three question types: demographic, behavioural and opinion. For every question, each respondent could give consistent answers between surveys, they could report a sensitive response in Natsal-3 but not in the web, or they could report a sensitive response in the web but not in Natsal-3. McNemar tests were used to test for each outcome whether, compared with reporting in Natsal-3, the responses were systematically different in the web survey, i.e. whether the within-person differences were at random or indicated a mode effect causing systematically higher reporting of more sensitive responses in one survey.

We pooled responses across outcomes to present summary statistics for the proportions of responses not changing, changing from more to less sensitive, and from less to more sensitive. We then conducted a logistic regression analysis to calculate odds ratios (ORs) for the effects of participant sex, question type (opinion or behaviour) and Natsal-3 mode (CAPI or CASI) on whether there was a change in reporting from Natsal-3 to the web survey, and (if there was) whether the change was from less to more sensitive rather than the converse. We included these variables to establish, firstly, whether the mode which is more similar to the web (i.e. CASI) produced significantly fewer answer differences, and, secondly, whether men and women were differently impacted by the change in mode (as previously mentioned). The regression models were fitted using generalised estimating equations to acknowledge the ‘clustering’ of outcomes by respondents. The OR’s were adjusted for the other variables in the model to account for the uneven spread of opinion and behaviour questions across CAPI or CASI.

We examined the number of differences in responses across the 31 behavioural and opinion questions reported by individuals. To examine the extent to which differences are correlated within individuals, we fitted a random effects model to the outcome (change or no change) pooling across questions, with random intercepts for individuals and fixed effects for which question was considered. The intra-individual correlation can then be calculated from the variance of the random effects, on the log-odds scale.

All analysis was conducted in Stata 13.

## Results

Of the 2440 Natsal-3 respondents invited, 527 completed the web survey (21.6%). Data from Natsal-3 show that, relative to non-respondents, those who took part in the follow-up were more likely to have higher educational qualifications, higher ranking jobs, to own their home and to be full-time students (data not shown). The characteristics of the web respondents are in S1 Table.

Table 1 shows responses to demographic questions, which are not deemed to be sensitive (with the possible exception of sexual identity). The large majority of respondents gave the same answer at the web survey.

**Table 1:** Demographic questions: differences in response.

	Differences in response:			
Men (N = 202)	Yes in web, no in Natsal-3	No in web, yes in Natsal-3	No difference	McNemar test p-value
Tenure–renting	2.5%	4.5%	93.0%	0.42
Ethnicity—non-white	0.0%	0.0%	100.0%	1.00
Economic activity—not in employment	3.5%	5.4%	91.1%	0.48
Household size– 2+ persons	3.0%	3.0%	94.1%	1.00
Highest education qualification—below degree level	1.7%	4.6%	93.7%	0.23
Sexual identity—not exclusively heterosexual	0.0%	0.0%	100.0%	1.00
**Women (N = 325)**				
Tenure–renting	4.7%	3.7%	91.6%	0.70
Ethnicity—non-white	0.0%	0.0%	100.0%	1.00
Economic activity-not in employment	5.8%	5.2%	88.9%	0.87
Household size– 2+ persons	1.8%	2.2%	96.0%	1.00
Highest education qualification—below degree level	0.6%	5.5%	93.9%	<0.001
Sexual identity—not exclusively heterosexual	1.8%	0.0%	98.2%	0.03

**Fig 3:**
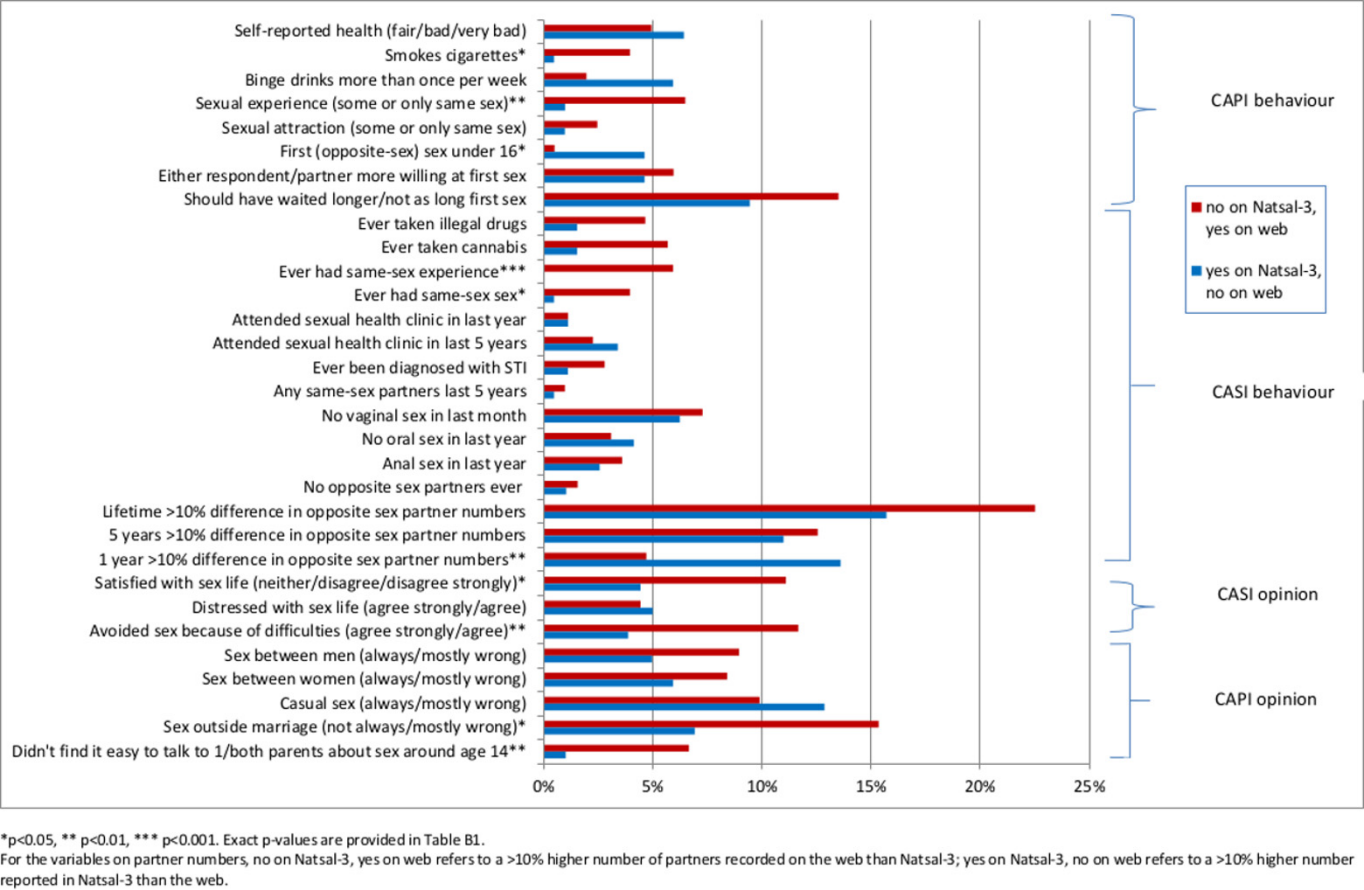
Men: Differences in response.

Figs 3 (men) and 4 (women) show the percentage of changed responses between surveys across individual behaviour and opinion questions; asterisks highlight a significant systematic mode effect. The percentages, estimates from each survey, and p-values are in S2 Table (men) and S3 Table (women).

**Fig 4:**
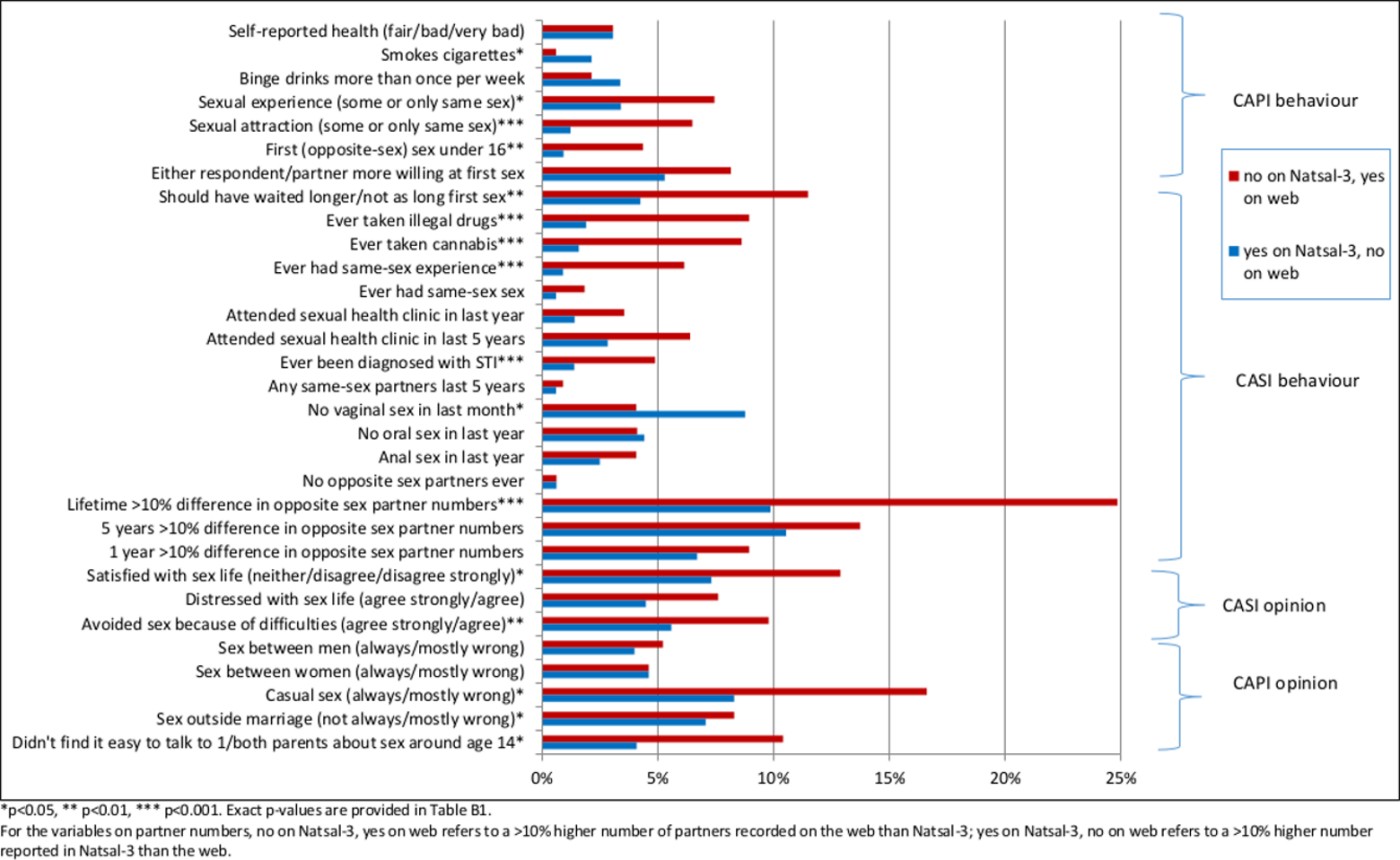
Women: Differences in response.

Significant mode effects can be seen for 9 questions (out of 31) for men and 12 for women. Where there were significant differences, the majority were in the predicted direction of higher rates of disclosure in the web survey of sensitive behaviours or socially censured views. These include higher reporting of same-sex experiences for men and women, and higher reporting of sexual debut aged under 16 for women but lower reporting for men. For women there was also higher reporting in the web of ever diagnosed with STI and no vaginal sex in the last month. Notable for women was the higher reporting in the web of the number of lifetime partners. The opposite was found for men, who were significantly more likely to report higher numbers of partners in the last year in Natsal-3 than on the web (but this was not found for partners in the last 5 years or lifetime.) Significant effects were evident for other sensitive behaviours, such as illegal drug use (for women). We found significant effects for several opinion questions, including satisfaction with current sex life, the acceptability of casual sex (for women) and of sex outside marriage (for men).

However, the prevalence estimates for the majority of questions for Natsal-3 and the web survey are similar (S2 and S3 Tables). Even where there is a significant mode effect, it does not necessarily indicate an important difference in the estimate between surveys; for example, among men, despite the clear mode effect, the estimated prevalence of first sex before age 16 years is 20.9% in Natsal-3 and 17.4% in the web survey.

The pooled proportions of differences in response by question type and Natsal-3 mode show that over 90% of responses were consistent between Natsal-3 and the web, with about 6% reporting more sensitive answers in the web survey, and about 3.5% reporting more sensitive answers in Natsal-3 (Table 2). The pooled responses were similar for men and women.

**Table 2:** Differences in response, pooled across questions, by question mode in Natsal-3.

	Differences in response:		
Men (N = 202)*	**Yes in web, no in Natsal-3**	**No in web, yes in Natsal-3**	**No difference**
CAPI behaviour (8 questions)	4.7%	4.0%	91.3%
CASI behaviour (15 questions)	4.4%	5.4%	90.2%
CASI opinion (3 questions)	9.1%	4.4%	86.5%
CAPI opinion (5 questions)	10.5%	5.8%	83.7%
All (31 questions)	5.9%	5.0%	89.1%
Women (N = 325)*	**Yes in web, no in Natsal-3**	**No in web, yes in Natsal-3**	**No difference**
CAPI behaviour (8 questions)	5.4%	2.6%	92.0%
CASI behaviour (15 questions)	5.2%	5.3%	89.5%
CASI opinion (3 questions)	10.1%	5.8%	84.1%
CAPI opinion (5 questions)	7.4%	7.3%	85.3%
All (31 questions)	6.1%	5.0%	99.96%

*Bases shown are for the full sample, but bases may vary for individual questions.

In Table 3 on the left, are adjusted ORs with 95% confidence intervals (CIs) which show that differences between modes were comparable for men and women, but were more likely for Natsal-3 questions asked in CASI (than CAPI). On the right of Table 3, the adjusted ORs demonstrate that, given a change in response between modes, the likelihood of a change from a less sensitive response in Natsal-3 to a more sensitive response in the web survey was unrelated to respondent sex, but was more likely for opinions (than behaviours) and somewhat less likely for CASI (than CAPI) questions in Natsal-3.

**Table 3:** Associations with reporting different answers, and if different answer, for reporting more sensitive response in the web and not in Natsal-3.

	Whether different answer			If different answer: Yes in web, no in Natsal-3		
	Adjusted Odds Ratio^1^	95% CI	p-value	Adjusted Odds Ratio^a^	95% CI	p-value
Men	1.00			1.00		
Women	1.00	(0.99–1.02)	0.94	1.01	(0.95–1.06)	0.78
Behaviour	1.00			1.00		
Opinion	1.06	(1.05–1.07)	0.00	1.06	(1.00–1.11)	0.04
CAPI	1.00			1.00		
CASI	1.01	(1.00–1.02)	0.01	0.95	(0.91–1.00)	0.06

^**a**^Odds ratio adjusted for other variables in the table.

Three in four (74.0%) respondents were inconsistent for, at most, only a few responses (between 0 and 4 differences out of 31 questions). The intra-individual correlation for differences across questions was 0.123, which suggests that respondents who changed one answer were somewhat more likely than other respondents to change another.

## Discussion

Our study aimed to assess the extent to which mode–CAPI/CASI versus web-based administration–might affect responses in a survey focused on sensitive behaviours and opinions. A mode effect was evident for some sensitive questions, but not for the majority, suggesting that the greater anonymity afforded by the web will not necessarily lead to higher levels of disclosure. The large majority (over 90%) of responses did not change across modes, and the vast majority of respondents gave inconsistent answers to no more than a handful of questions. However, there were significant mode effects for about one-third of the questions, generally leading to more reporting of sensitive behaviours and less socially desirable opinions in the web survey than in Natsal-3.

There are limitations to our study. Rather than all Natsal-3 interviews taking place before the web survey, ideally a random half of the sample would have completed the web survey first to minimise factors contaminating what we can conclude about the effects of mode directly (which may include genuine changes over time in some variables). Another limitation is the low response rate (21.6%) which suggests that the web respondents may be particularly interested in the survey topic and possibly not representative of the whole population. In this study evidence for mode effects comes from a comparison of responses from two modes in the same individuals and so representativeness of the achieved sample is less important than it would be if the two modes were offered to different individuals. Nevertheless a low response rate limits the extent to which we can be confident these results would be found throughout the population. Also, the web survey included only a sub-set of Natsal-3 questions, so the context for some questions, even though identically worded, may have been somewhat different. Another limitation is that analyses requiring pooling of outcomes across questions obliged us to specify the ‘more sensitive’ response, which can be difficult to do objectively. A particular strength of our study was that we were able to assess within-person change, which differs from most studies on mode effects.[28–30]

There were differences between Natsal-3 and the web survey regarding the presentation of the ‘don’t know’ category for the CAPI opinion questions. For these, ‘don’t know’ was not included on the show cards used in Natsal-3 (although respondents could spontaneously give ‘don’t know’ as an answer), but it was shown on the screen as a response category in the web survey. Although this resulted in more respondents selecting ‘don’t know’ in the web survey, the differences for these questions showed a consistent pattern with less socially desirable answers being more frequently selected in the web survey.

Consistent with expectations, for demographic questions, there was very little or no change between modes. Although not all the differences for sensitive CAPI questions were statistically significant, they were all in the direction expected, even when that differed for men and women. For example, men were more likely to report their first sexual experience being under age 16 in Natsal-3 than in the web survey, while the opposite was found for women, reinforcing the view that men are more likely to exaggerate their sexual experiences and women are more likely to downplay theirs.[27,31,32]

Our findings apply beyond sexual health research, as differences were found in reports of other behaviours (e.g. smoking, drinking, drug use). Reports of illegal drug use appear to be under-reported in Natsal-3 despite (arguably) this being one of the less sensitive questions in the survey.[33] Conversely, some of the seemingly more sensitive items show less change (e.g. STI diagnoses, experience of anal sex), underlining the complexities of mode effect, and perhaps highlighting the importance of context. Respondents may be more willing to report sensitive sexual behaviours because of their obvious relevance to a sexual health survey, whereas the relevance of drug use in such a survey may not be apparent.

We found high levels of consistency across modes, with only a small minority of respondents taking advantage of the greater privacy offered by the web to disclose sensitive behaviours or opinions that they did not mention in CAPI/CASI. This suggests that a well-designed CAPI/CASI survey, which provides robust reassurances of confidentiality, is able to elicit high quality data when measuring sensitive behaviours. Having said that, in line with previous studies, we did obtain slightly higher reports of some sensitive behaviours when using the web, which suggests there may be an advantage of using this mode for surveys on sensitive issues.

While web surveys are now the norm in market research, this is not the case for academic or government research due to concerns over data quality. Difficulties remain over sampling, as there is no cost-effective means of obtaining a probability sample for web surveys of the general population. Market research makes extensive use of the large volunteer web panels maintained by survey organisations. However, as previous research has shown, while the use of a web panel to conduct a general population survey such as Natsal may result in higher reports of some sensitive behaviours (e.g. same-sex experience), the usefulness of the data would be questionable given the significant bias generally found when using volunteer panels.[25,34] But collecting survey data from randomly sampled members of a probability-based panel (see, for example, [34]) or from the same respondents recruited with probability-based methods to answer in another mode, as was done here, may have the potential to improve the quality of responses.

There is increasing interest in academia and government to make greater use of data collection via the web in settings where web coverage is high. The impetus is more for using the web as part of a mixed mode design rather than carrying out a stand-alone web survey, mainly because of the data quality issues described above. There are different approaches to mixing modes, but they generally involve either: a) offering respondents a choice between modes to encourage higher response [35,36] or b) changing modes during different stages of data collection for a repeated cross-sectional survey [37] or for a longitudinal study.[38,39] For example, the UK’s ‘Understanding Society’, a panel of 40,000 households, has carried out experiments on mixing modes within and between waves of data collection.[40] Our findings suggest that potential mode effects are likely to vary by question type and content, as well as with the particular mix of modes used. While there may be potential for reducing coverage error, mixing modes within a survey may change the mix of measurement error which may impact on comparisons between sub-groups or looking at trends over time.[41,42] Researchers wishing to adopt a mixed mode survey, therefore, will need to undertake careful development work to try to minimise these effects, and to understand where they are likely to arise, and whether and how such effects can be accounted for during analysis.

## Supporting Information

S1 Table Distributions of socio-demographic characteristics for web follow-up respondents, by sex of respondent. (DOCX)

S2 Table Men: Key behaviours and opinions: distributions, differences in response, and p-values. (DOCX)

S3 Table Women: Key behaviours and opinions: distributions, differences in response, and p-values. (DOCX)
