# Supplementary material for: Using the Web to Collect Data on Sensitive Behaviours: A Study Looking at Mode Effects on the British National Survey of Sexual Attitudes and Lifestyles
Source: PLoS One. 2016 Feb 11;11(2):e0147983. doi: 10.1371/journal.pone.0147983 (PMC4750932; doi:10.1371/journal.pone.0147983)
Supplement: S1 Table — (DOCX) [file pone.0147983.s001.docx]

**S1 Table: Distributions of socio-demographic characteristics for web follow-up respondents, by sex of respondent**

|  | **Men** | **Women** |
| --- | --- | --- |
| **Age group** |  |  |
| 16-24 | 24.8% | 30.8% |
| 25-34 | 22.8% | 28.9% |
| 35-44 | 9.4% | 14.2% |
| 45-54 | 12.9% | 11.1% |
| 55-64 | 19.8% | 8.6% |
| 65-74 | 10.4% | 6.5% |
| **Highest educational qualification** |  |  |
| Degree level or higher | 36.1% | 30.2% |
| A-Levels or equivalent | 16.8% | 27.4% |
| O-Level/other | 37.1% | 33.9% |
| None | 9.9% | 8.6% |
| **Tenure** |  |  |
| Own outright | 31.3% | 23.2% |
| Buying with mortgage/loan | 38.8% | 41.2% |
| Shared ownership (pays part rent/part mortgage) | 1.0% | 1.2% |
| Rents | 23.9% | 30.3% |
| Lives rent free | 5.0% | 4.0% |
| **Sexual identity** |  |  |
| Heterosexual/straight | 94.1% | 96.6% |
| Gay/lesbian | 4.0% | 1.5% |
| Bisexual | 2.0% | 1.9% |
| Other | 0.0% | 0.0% |
| **Household size** |  |  |
| 1 person | 20.8% | 14.2% |
| 2 persons | 39.6% | 28.6% |
| 3 persons | 15.8% | 21.9% |
| 4+ persons | 23.8% | 35.4% |
| **NS-SEC** |  |  |
| Managerial & professional occupations | 43.1% | 33.9% |
| Intermediate occupations | 13.4% | 19.7% |
| Semi-routine/routine occupations | 17.3% | 19.4% |
| No job | 6.4% | 6.2% |
| Student in full-time education | 19.3% | 20.3% |
| Not classifiable | 0.5% | 0.6% |
