# Supplementary material for: Using the Web to Collect Data on Sensitive Behaviours: A Study Looking at Mode Effects on the British National Survey of Sexual Attitudes and Lifestyles
Source: PLoS One. 2016 Feb 11;11(2):e0147983. doi: 10.1371/journal.pone.0147983 (PMC4750932; doi:10.1371/journal.pone.0147983)
Supplement: S2 Table — (DOCX) [file pone.0147983.s002.docx]

**S2 Table: Men: Key behaviours and opinions: distributions, differences in response, and p-values**

|  | **Natsal-3 estimate** | **Yes in web,**  **no in Natsal-3** | **No in web,**  **yes in Natsal-3** | **No difference** | **Web estimate** | **p-value** |
| --- | --- | --- | --- | --- | --- | --- |
| Self-reported health (fair/bad/very bad) | 11.4% | 6.4% | 5.0% | 88.6% | 9.9% | 0.10 |
| Smoking status | 15.4% | 0.5% | 4.0% | 95.5% | 18.8% | 0.04 |
| Binge drinks more than once per week | 14.4% | 5.9% | 2.0% | 92.1% | 10.4% | 0.08 |
| Sexual experience (some or only same sex) | 10.4% | 1.0% | 6.5% | 92.5% | 16.0% | 0.01 |
| Sexual attraction (some or only same sex) | 9.9% | 1.0% | 2.5% | 96.5% | 11.4% | 0.45 |
| First (opposite-sex) sex aged under 16 | 20.9% | 4.6% | 0.5% | 94.8% | 17.4% | 0.02 |
| Either respondent/partner more willing at first sex | 7.3% | 4.6% | 6.0% | 89.4% | 8.6% | 0.80 |
| Should have waited longer/not as long at first sex | 23.3% | 9.5% | 13.5% | 77.0% | 29.7% | 0.39 |
| Ever taken illegal drugs | 39.4% | 1.6% | 4.7% | 93.8% | 40.6% | 0.15 |
| Ever taken cannabis | 37.3% | 1.6% | 5.7% | 92.7% | 39.6% | 0.06 |
| Ever had a same-sex experience | 11.4% | 0.0% | 5.9% | 94.1% | 17.3% | 0.00 |
| Ever had same-sex sex | 9.9% | 0.5% | 4.0% | 95.5% | 13.4% | 0.04 |
| Attended a sexual health clinic in last year | 5.0% | 1.1% | 1.1% | 97.7% | 5.6% | 1.00 |
| Attended a sexual health clinic in last 5 years | 10.8% | 3.4% | 2.3% | 94.3% | 10.1% | 0.75 |
| Ever been diagnosed with an STI | 11.4% | 1.1% | 2.8% | 96.1% | 13.8% | 0.45 |
| Any same sex partners in last 5 years | 5.9% | 0.6% | 1.1% | 98.3% | 6.4% | 1.00 |
| No vaginal sex in last month | 46.8% | 6.3% | 7.3% | 86.5% | 46.6% | 0.85 |
| No oral sex in last year | 34.3% | 4.1% | 3.1% | 92.7% | 38.1% | 0.79 |
| Anal sex in last year | 7.5% | 2.6% | 3.6% | 93.8% | 12.8% | 0.77 |
| No opposite sex partners ever | 11.0% | 1.0% | 1.6% | 97.4% | 9.9% | 1.00 |
| Satisfied with sex life (neither/disagree/disagree strongly) | 43.5% | 4.4% | 11.1% | 84.4% | 50.8% | 0.04 |
| Distressed with sex life (agree strongly/agree) | 11.9% | 5.0% | 4.4% | 90.6% | 12.0% | 1.00 |
| Avoided sex because of difficulties (agree strongly/ agree) | 9.5% | 3.9% | 11.7% | 84.4% | 17.5% | 0.01 |
| Sex between men (always/mostly wrong) | 32.8% | 5.0% | 9.0% | 86.1% | 36.6% | 0.18 |
| Sex between women (always/mostly wrong) | 27.2% | 5.9% | 8.4% | 85.6% | 29.7% | 0.46 |
| Casual sex (always/mostly wrong) | 38.1% | 12.9% | 9.9% | 77.2% | 41.1% | 0.46 |
| Sex outside marriage (not always/mostly wrong) | 19.3% | 6.9% | 15.3% | 77.7% | 27.7% | 0.02 |
| Didn't find it easy to talk to one/both parents about sex around age 14 | 12.8% | 1.0% | 6.7% | 92.3% | 93.4% | 0.01 |
