# Supplementary material for: Using the Web to Collect Data on Sensitive Behaviours: A Study Looking at Mode Effects on the British National Survey of Sexual Attitudes and Lifestyles
Source: PLoS One. 2016 Feb 11;11(2):e0147983. doi: 10.1371/journal.pone.0147983 (PMC4750932; doi:10.1371/journal.pone.0147983)
Supplement: S3 Table — (DOCX) [file pone.0147983.s003.docx]

**S3 Table: Women: Key behaviours and opinions: distributions, differences in response, and p-values**

|  | **Natsal-3 estimate** | **Yes in web,**  **no in Natsal-3** | **No in web,**  **yes in Natsal-3** | **No difference** | **Web estimate** | **p-value** |
| --- | --- | --- | --- | --- | --- | --- |
| Self-reported health (fair/bad/very bad) | 13.2% | 3.1% | 3.1% | 93.8% | 13.2% | 0.00 |
| Smoking status | 16.3% | 2.2% | 0.6% | 97.2% | 17.9% | 0.18 |
| Binge drinks more than once per week | 6.2% | 3.4% | 2.2% | 94.5% | 4.9% | 0.48 |
| Sexual experience (some or only same sex) | 15.4% | 3.4% | 7.5% | 89.1% | 19.5% | 0.04 |
| Sexual attraction (some or only same sex) | 15.4% | 1.2% | 6.5% | 92.3% | 20.7% | 0.00 |
| First (opposite-sex) sex aged under 16 | 20.1% | 0.9% | 4.4% | 94.7% | 23.7% | 0.13 |
| Either respondent/partner more willing at first sex | 20.1% | 5.3% | 8.2% | 86.5% | 22.4% | 0.29 |
| Should have waited longer/not as long at first sex | 28.4% | 4.3% | 11.5% | 84.3% | 35.0% | 0.01 |
| Ever taken illegal drugs | 31.6% | 1.9% | 8.9% | 89.1% | 37.5% | 0.00 |
| Ever taken cannabis | 30.0% | 1.6% | 8.6% | 89.8% | 36.0% | 0.13 |
| Ever had a same-sex experience | 85.5% | 0.9% | 6.2% | 92.9% | 19.7% | 0.00 |
| Ever had same-sex sex | 9.2% | 0.6% | 1.8% | 97.5% | 10.5% | 0.29 |
| Attended a sexual health clinic in last year | 5.6% | 1.4% | 3.6% | 95.0% | 8.7% | 0.18 |
| Attended a sexual health clinic in last 5 years | 19.2% | 2.8% | 6.4% | 90.7% | 22.2% | 0.08 |
| Ever been diagnosed with an STI | 19.7% | 1.4% | 4.9% | 93.7% | 23.9% | 0.00 |
| Any same sex partners in last 5 years | 4.9% | 0.6% | 0.9% | 98.5% | 4.6% | 1.00 |
| No vaginal sex in last month | 46.4% | 8.8% | 4.1% | 87.1% | 41.1% | 0.03 |
| No oral sex in last year | 32.2% | 4.4% | 4.1% | 91.5% | 33.7% | 1.00 |
| Anal sex in last year | 8.1% | 2.5% | 4.1% | 93.4% | 12.8% | 0.38 |
| No opposite sex partners ever | 11.4% | 0.6% | 0.6% | 98.7% | 11.0% | 1.00 |
| Satisfied with sex life (neither/disagree/disagree strongly) | 40.7% | 7.3% | 12.9% | 79.8% | 44.6% | 0.05 |
| Distressed with sex life (agree strongly/agree) | 10.3% | 4.5% | 7.6% | 87.9% | 13.8% | 0.18 |
| Avoided sex because of difficulties (agree strongly/ agree) | 12.3% | 5.6% | 9.8% | 84.6% | 16.9% | 0.10 |
| Sex between men (always/mostly wrong) | 14.8% | 4.0% | 5.2% | 90.8% | 16.0% | 0.58 |
| Sex between women (always/mostly wrong) | 15.4% | 4.6% | 4.6% | 90.8% | 15.4% | 1.00 |
| Casual sex (always/mostly wrong) | 50.8% | 8.3% | 16.6% | 75.1% | 42.5% | 0.00 |
| Sex outside marriage (not always/mostly wrong) | 12.9% | 7.1% | 8.3% | 84.6% | 14.2% | 0.67 |
| Didn't find it easy to talk to one/both parents about sex around age 14 | 22.0% | 4.1% | 10.4% | 85.5% | 84.4% | 0.00 |
